# Difficult Ventilation in an Infant After Successful Intubation

**DOI:** 10.5152/TJAR.2021.21191

**Published:** 2022-08-01

**Authors:** Kübra Evren Şahin, Canan Salman Önemli

**Affiliations:** Department of Anaesthesiology and Reanimation, Dr. Behçet Uz Children’s Hospital, İzmir, Turkey

**Keywords:** Airway obstruction, endotracheal tube connector, manufacturing defect

## Abstract

Manufacturing defects in the connector of the endotracheal tube are not frequently encountered in emergency and planned intubations. In cases where they are encountered, however, they may cause partial or complete airway obstruction in sick infants with limited reserves, giving rise to a life-threatening situation following the intubation. For this reason, endotracheal tubes must be checked carefully before use. To this end, a stylet can be passed through the tube to check for a defect in the tube lumen or tube connector as part of a routine check of an endotracheal tube. This report features a patient who experienced a complete airway obstruction following intubation due to a manufacturing defect in the connector of the endotracheal tube.

**Main Points**

- Control of the endotracheal tube is important during the preparation for intubation.
- Should check the cuff of the endotracheal tube by inflating it with a syringe.
- Visual inspection of the endotracheal tube connector and the lumen of the endotracheal tube is also vital.

## Introduction

Endotracheal intubation is crucial in securing the patency of the airway in cases where spontaneous breathing is not sufficient. The breathing machine to be connected to the patient, the breathing circuit, and the lumen and connector of the endotracheal must be checked before an intubation attempt taking into account that failure to ventilate the patient following endotracheal intubation may give rise to a life-threatening situation.

The inability to ventilate the patient after successful endotracheal intubation may be due to various causes that are related to the patient (pneumothorax, bronchospasm, chest wall rigidity, endobronchial mass), the endotracheal tube (kinking of the tube, obstruction in the tube lumen), and the breathing circuit (detachment in the breathing circuit, obstruction in the breathing circuit).^1,2^ This report features a paediatric patient who could not be ventilated following endotracheal intubation due to a total obstruction in the airway due to a manufacturing defect in the connector of the endotracheal tube.

## Case Presentation

**Figure 1.**
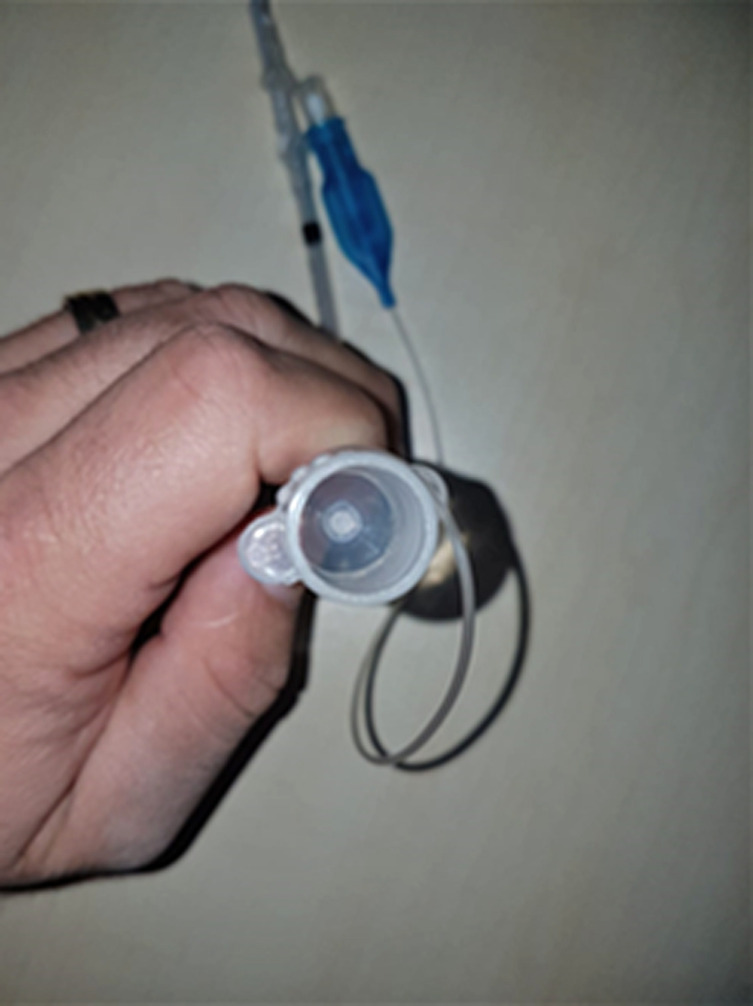
Endotracheal tube connector lumen was completely closed.

A 67-day-old male infant weighing 4100 g and diagnosed with transcobalamin defect, metabolic syndrome, ventriculomegaly, hydrocephalus, hypertension, patent foramen ovale, atrial septal defect, and refractory epilepsy was scheduled for ventriculoperitoneal shunt insertion by neurosurgeons under general anaesthesia. Physical examination of the patient before the surgery did not reveal an upper respiratory tract infection or breathing difficulty. The pre-anaesthetic assessment was compatible with the American Society of Anesthesiologists (ASA) III classification. Accordingly, the vital signs were within normal ranges, the mouth opening was sufficient, and the thyromental distance was 2 cm. The patient was moved to the operating room after 6-hour fasting. An electrocardiogram was taken, and non-invasive blood pressure and peripheral oxygen saturation were monitored. The patient was administered a mixture of sevoflurane and oxygen via a face mask. A venous line was installed on the dorsum of the hand using a 24-G intravenous cannula under inhalation anesthesia. The anaesthesia was induced by the administration of 0.1 mg kg^-1^ midazolam, 1 μg kg^-1^ fentanyl, and 0.5 mg kg^-1^ rocuronium. The patient was intubated using a cuffed endotracheal tube size 3. However, no air entry was observed into the lungs or even the stomach while manually ventilating the patient using the anaesthesia balloon. The endotracheal tube was withdrawn, and the ventilation was continued with an anaesthesia mask since no air entry into the lungs and the stomach was heard on auscultation, normal capnography waveform was absent, and there was an increase in peak airway pressure. The endotracheal tube was examined after its removal. It was decided to pass a stylet through the endotracheal tube to understand whether there was a defect in the tube lumen. Consequentially, it was realized while attempting to pass a stylet through the endotracheal tube that the connector of the endotracheal tube was totally obstructed (Figure 1). Therefore, the patient was re-intubated using another cuffed endotracheal tube size 3. Upon ventilation of the patient by the anaesthesia balloon, air entry into the lungs and capnogram waveform was observed, and the tube was anchored at 10 cm while both lungs were equally ventilated. In this way, no problems occurred in the intraoperative and postoperative periods.

## Discussion

Endotracheal tube defect causes resistance to the inflation of the anesthesia balloon in manual ventilation and leads to a constant increase in the inspiratory pressures after successful endotracheal intubation.^3^ The defects in the endotracheal tube connector may cause a partial^4-10^ or a complete airway obstruction.^3,11^ A thorough review of the literature revealed that the endotracheal tube was checked inside the mouth using a laryngoscope,^3,4,9^ aspiration of the tube lumen,^3-5,7,8,10^ and extubation and re-intubation^9-11^ in patients who could not be ventilated sufficiently following successful intubation. The repetition of such invasive procedures can be avoided by a detailed examination of the endotracheal tube (Table 1).

The manufacturing defects in the endotracheal tubes are not common and may thus be overlooked during an examination before an intubation attempt. There was a metallic flap narrowing the tube lumen when the connector of the endotracheal tubes used to be manufactured from aluminum, and checking the endotracheal tube connecter would have been recommended before the intubation attempt.^12^ Interestingly, there seems to be only one paediatric case report in which the defect in the endotracheal tube connector was noticed during a check performed before the intubation. The other endotracheal tube connecter defects reported in the literature have been detected only after performing the intubation procedure. It must be kept in mind that difficulty in ventilating despite successful intubation can be lifethreatening in paediatric patients with a poor overall condition requiring emergency intubation.

## Conclusion

The cuff of the endotracheal tube is routinely checked before intubation. However, given the gravity of the complications that may arise in relation to the endotracheal tube defects, passing a stylet through the endotracheal tube in order to check whether there is a defect in the tube lumen and/or the tube connector may be included in the routine check of the endotracheal tube.

## Figures and Tables

**Table 1.** Defects in the Endotracheal Tube Connector in the Literature

**References**	**Age of Patient**	**Operation**	**Endotracheal Tube Connector (ETTc) Defect Site**
Evren Sahin et al	67 days	Ventriculoperitoneal shunt	Completely obstructed ETTc
Praneeth J et al^3^	12 years	Bilateral adenotonsillectomy	Completely obstructed ETTc
Dwivedi et al^4^	3 months	Pyeloplasty	Pinhole opening in an ETTc
Sethi et al^5^	3 months	Emergency arthrotomy	The narrow lumen ETTc
Kumar et al^6^	5 months	Cleft lip and palate repair	The narrow lumen ETTc
Jain et al^7^	7 months	Bilateral inguinal herniotomy	The narrow orifice ETTc
Jain et al^8^	3 years	Percutaneous cystolithotripsy	Partial obstructed ETTc
Shamshery et al^9^	1 months	Pyloromyotomy	Obliteration in the ETTc
Malde et al^10^	2 years	Laparotomy	Membranous diaphragm in the distal end of the ETTc
Singhal et al^11^	8 months	Inguinal herniotomy	Completely obstructed ETTc
